# Supplementary material for: Epidemiology of hepatitis B virus co-infection among people living with HIV in four countries in sub-Saharan Africa
Source: PLoS One. 2026 May 27;21(5):e0337789. doi: 10.1371/journal.pone.0337789 (PMC13215549; doi:10.1371/journal.pone.0337789)
Supplement: Attachment — Submitted filename: Response to Reviewers.docx [file pone.0337789.s003.docx]

**Response to Reviewers**

| Comment | Response |
| --- | --- |
| Reviewer #1 | |
| Use of a single marker (HBsAg), as a limitation | We acknowledge this important point and have revised the limitations section to note that reliance on a single marker (HBsAg) could have led to rare false positives from resolved infection and missed occult cases, especially in patients on ART. The word ‘markers’ has been corrected to marker’ as guided. |
| Reviewer #2 (ScreenIT Report) | |
| Inclusion/Exclusion Criteria | We have now added a clear description of the inclusion and exclusion criteria in the Methods section. Participants aged ≥15 years with and without HIV who had a valid HBV test result at enrollment were included. Participants without a valid HBV test were excluded from the analysis. |
| Flow Chart and Attrition | A CONSORT-style flow diagram has been added to the manuscript, as Figure 1, to illustrate participant enrollment, exclusions, and analytic subgroups. Attrition is minimal, with only 49 participants excluded due to missing HBV test results. |
| Randomization | This was an observational cohort study without randomization. |
| Blinding | As this was an observational study, blinding was not applicable. |
| Power Analysis | A formal power analysis was not conducted as this was a secondary analysis of existing cohort data. We have added a statement to clarify this in the Methods section. |
| RRIDs | We have now included RRID identifiers where applicable. |
| Open Code | As a matter of policy, we do not share code used for analysis in public repositories. |
| Open Data | While the full dataset cannot be made publicly available due to ethical restrictions, a minimal de-identified dataset is available upon request from the Data Coordinating and Analysis Center, as described in the Data Availability Statement. |
